# Distinct expression patterns of mitochondrially localized YFP in neuronal subsets in the retina of three transgenic mouse lines

**DOI:** 10.1186/1756-0500-3-253

**Published:** 2010-10-06

**Authors:** Robert W Burgess, Peter G Fuerst

**Affiliations:** 1 The Jackson Laboratory, Bar Harbor, ME 04609, USA; 2 Department of Biological Sciences and WWAMI Medical Education Program, The University of Idaho, Moscow ID 83844, USA

## Abstract

**Background:**

Transgenic labels that allow the visualization of specific populations of neurons have proven to be powerful tools for research. Further developing such resources to label additional cell types and specific organelles within these cell will provide additional experimental opportunities.

**Findings:**

The retinal expression profile of a mitochondria-localized yellow fluorescent protein (YFP) in each of three transgenic mouse lines was determined. Each line, Mito-R, Mito-Y and Mito-Z, expresses YFP in distinct and reproducible populations of retinal neurons. In the Mito-R line, YFP is expressed in most or all retinal ganglion cells (RGCs) and photoreceptors making this line useful for studying axonal transport in diseases such as glaucoma and photoreceptor degeneration related to transport of mitochondria into the inner segments. In the Mito-Y line, YFP is expressed in many cell types in the dorsal retina and in a rough mosaic population of RGCs in the rest of the retina, making this line useful for study of how retinal mosaics are organized. In the Mito-Z line, YFP is expressed in a subset of RGCs, amacrine cells, bipolar cells and photoreceptors. The Mito-Z line is inserted on the X-Chromosome, resulting in X-inactivation mosaicism in female mice carrying a single copy of the transgene. In the female hemizygous retina, expression is present in distinct clonal columns, making this transgenic line useful for analysis of clonal proliferation and lateral migration of retinal neurons.

**Conclusion:**

The retinal expression profiles of three transgenic mouse lines that express a mitochondrially localized YFP were characterized in this study. These lines will allow researchers to isolate and identify cell types within the retina and to study retinal mitochondrial trafficking and disease.

## Introduction

The retina contains at least fifty-five distinct types of neurons that interact to form the functional circuitry of vision [1,2]. Retinal cell types are defined by a mixture of physiology, morphology, stratification of processes and antigenic markers [2-4]. Transgenic mouse strains that express fluorescent proteins have greatly enhanced understanding of the development and function of various populations of retinal neurons [3,5-7]. To further the development of such resources, three transgenic mouse lines that express YFP fused to the cytochrome oxidase 8 (COX8) mitochondrial localization sequence, under control of the Neuron Specific Enolase (Eno2) promoter, were previously generated [8]. The *Eno2 *neural promoter used to drive expression of YFP in these transgenic lines is sensitive to position effect related to the genomic insertion site of the transgene, similar to other transgenic strains [9]. In this study the retinal expression profile of YFP was assayed for each line. Each transgenic line expresses YFP in a distinct and reproducible set of retinal neurons, making each useful for different applications related to retinal biology.

## Results

Three strains of transgenic mice expressing YFP fused to the COX8 mitochondrial localization sequence and under control of the *Eno2 *promoter were generated by pronuclear injection as previously described [8]. Each strain was found to express YFP in reproducible, discrete subpopulations of retinal neurons. Marker analysis and immunohistochemistry was performed to determine what cell populations express YFP in each transgenic line (Summarized in Table 1).

**Table 1:** Mito-YFP expression in retinal cell types

	Mito-Z	Mito-Y (central/non-dorsal peripheral)	Mito-Y (dorsal)	Mito-R
Ganglion Cells	++	+	++	+++
Smi-32 (+) Cells	++	-	+	+++
Amacrine Cells	++	-	+	+
Dopaminergic Amacrine Cells	+++	-	+	-
Cholinergic Amacrine Cells	-	-	-	-
Bipolar cells	+++	-	++	-
Horizontal Cells	+	-	++	+
Photoreceptors	+	-	+++	+++

**Figure 1:**
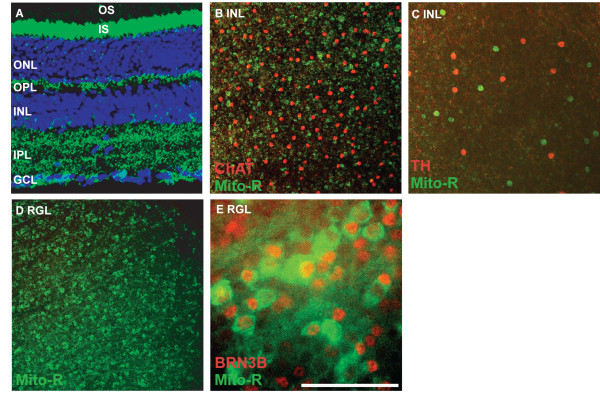
**YFP expression in the Mito-R retina**. **A**, YFP fluorescence was observed in all or nearly all photoreceptors, as well as horizontal cells and a subset of amacrine and ganglion cells. **B**, YFP in the inner nuclear layer (INL) does not colocalize with ChAT, a marker of starburst amacrine cells. **C**, YFP in the INL does not colocalize with tyrosine hydroxylase (TH), a marker of dopaminergic amacrine cells. **D**, YFP in the Mito-R retinal ganglion layer (RGL). E, Mito-R retina labeled with antibodies to BRN3b, a marker of RGCs. The majority of YFP positive cells are BRN3b positive. The scale bar in (**D**) is equivalent to 225 μm in **A **and 387.5 μm in **B **and **D**, 232.5 μm in **C **and 62.5 μm in **E**.

In the Mito-R line, YFP is expressed in retinal ganglion cells, as evidenced by the presence of YFP in retinal ganglion cell axons, some amacrine cells, horizontal cells and photoreceptors (Figure 1A and 1C and Table 1 N > 10). The retinal ganglion cell layer (RGL) also contains displaced amacrine cells, which may represent as many as half the cells present in this layer. To determine the extent to which YFP-labeled cells in the RGL were amacrine cells versus RGCs, the colocalization of YFP with the transcription factor BRN3b, a marker of approximately 80% of retinal ganglion cells, was assayed. The majority of BRN3b-positive cells were also YFP-positive, indicating that YFP is expressed in the majority of RGCs in the Mito-R retina (Figure 1E). YFP is not expressed in cholinergic or dopaminergic amacrine cells in the Mito-R retina, although other cells within the inner nuclear layer were YFP-positive (Figure 1B).

**Figure 2:**
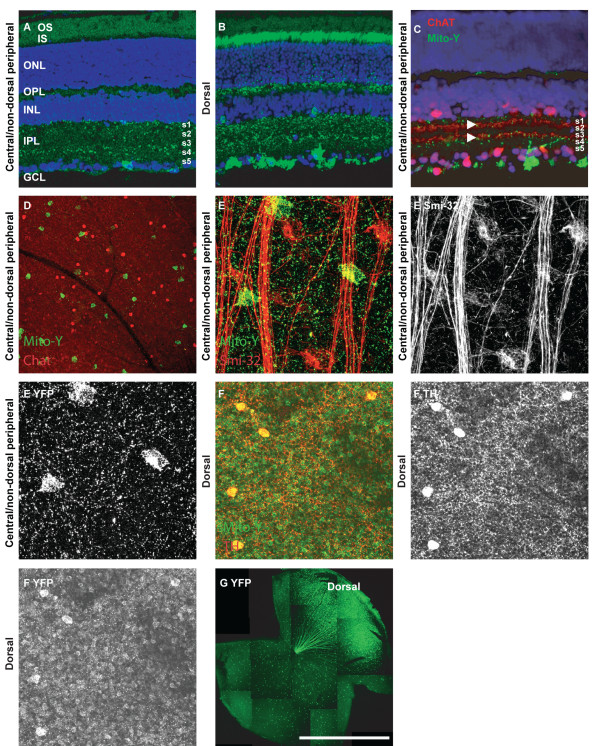
**YFP expression in the Mito-Y retina**. **A **and **B**, YFP fluorescence in sections in the central and non-dorsal peripheral (**A**) and dorsal (**B**) Mito-Y retina, respectively. YFP is localized to a small number of ganglion cells in the central and non-dorsal peripheral retina and to dendrites in S1, S2 and S4. In the dorsal retina, YFP is localized to all or nearly all photoreceptors, as well as horizontal cells and a subset of amacrine and ganglion cells. **C**, Section of central Mito-Y retina labeled with antibodies to ChAT. ChAT labels two bands in S2 and S4, proximal to YFP positive bands. **D**, Despite the proximity of cholinergic and YFP labeling in the Mito-Y inner nuclear layer, Mito-Y positive cells in the RGL are not cholinergic. **E**, Mito-Y positive cells in the dorsal and central or non-dorsal peripheral RGL are not non-phosphorylated neurofilament (Smi-32) immunopositive. **F**, Dopaminergic cells in the dorsal Mito-Y retina express YFP. **G**, Reconstructed montage of entire Mito-Y retina. The scale bar in **(G) **is equivalent to 225 μm in A-C and F, 387 μm in **D**, 66 μm in **E **and 1.45 mm in **G**.

In the Mito-Y line, YFP is differentially expressed in retinal domains (N > 10). In the dorsal retina, YFP is expressed in a large number of cell types compared to the rest of the retina (Table 1 and Figure 2G) [10]. In the dorsal retina YFP is expressed in a subpopulation of retinal ganglion, amacrine, bipolar and horizontal cells, as well as all or nearly all photoreceptors (Figure 2B and 2G). In the central and non-dorsal peripheral retina, YFP is expressed in a very small number of retinal ganglion cells, which are organized in a loose mosaic pattern (Figure 2A, G and Table 1) [10]. Colabeling sections of Mito-Y retina with ChAT was performed to label the ON-OFF cholinergic bands. YFP positive RGCs laminated dendrites adjacent to S2 and S4, and YFP expression was also observed in S1 (Figure 2C). These cells are not cholinergic amacrine cells, as evidenced by the lack of ChAT immunoreactivity, and do not overlap with non-phosphorylated neurofilament (Smi-32) positive alpha cells (Figure 2D and 2E). Dopaminergic cells are YFP-positive in the dorsal Mito-Y retina, but not other portions of the retina (Figure 2F and data not shown).

**Figure 3:**
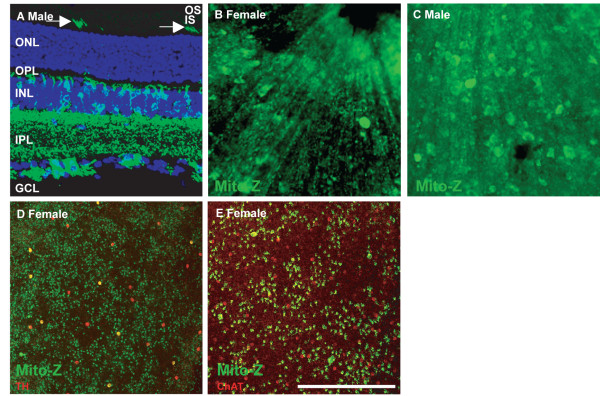
**YFP expression in the Mito-Z retina**. **A**, YFP is expressed in a subset of ganglion, amacrine and bipolar cells, as well as a very small number of photoreceptors (arrows). **B **and **C**, Retina from female and male mouse, respectively. Mito-Z labels patches of cells in the female retina (**B**) and throughout the male retina (**C**). **D**, Whole retina, from a female mouse, stained with antibodies to TH. YFP is expressed in roughly half of the retina. One half of dopaminergic amacrine cells in the female retina, which are all YFP positive in the male retina, are YFP positive. **E**, Mito-Z positive cells are not cholinergic. The scale bar in panel (**E) **is equivalent to 225 μm in **A**, 150 μm in **B **and **C**, and 387 μm in **D **and **E**.

In the Mito-Z line, YFP is expressed in a subset of retinal ganglion, amacrine, horizontal and bipolar cells as well as a small number of photoreceptors (Figure 3A N > 10). Based on the absence of male to male inheritance of the transgene, and exclusive male to female inheritance of the transgene, the Mito-Z line insertion site is on the X-Chromosome and is inactivated in roughly half of neurons in the retinas of hemizygous female mice (no transgenic male pups and all transgenic female pups out of over 100 pups from greater than twenty pairs consisting of Mito-Z hemizygous male mice crossed to wild type females) (Figure 3B and 3C) [11]. YFP expression overlapped with non-phosphorylated neurofilament, a marker of alpha retinal ganglion cells, and tyrosine hydroxylase (TH), a marker of dopaminergic amacrine cells, but not ChAT, a marker of starburst amacrine cells (data not shown; Figure 3C and Table 1).

## Discussion

Three transgenic mouse lines expressing mitochondrially localized YFP in different populations of retinal neurons are described in this study. YFP is expressed in distinct and reproducible cell populations in the retinas of each transgenic line, but the expression pattern is different for each transgenic line, Therefore, these strains are useful to researchers seeking to assay different aspects of retinal biology and disease.

### Study of Mitochondrial Diseases

The YFP transgene is fused to the mitochondrial localization signal of mouse COX8 (the N-terminal 34 amino acids). Defective mitochondrial transport within retinal neurons is speculated to underlie diseases of both retinal ganglion cells and retinal photoreceptors [12]. The differential expression of YFP in subsets or all RGCs and photoreceptors offers a tractable system by which researchers studying these processes can visualize mitochondria either *in vivo *or *in vitro*. Such approaches have been applied to motor neurons, and facilitate studies of mitochondrial morphology, trafficking, and fusion. In the eye, autosomal dominant optic atrophy 1 is caused by mutation of the *OPA1 *gene. OPA1 encodes a mitochondrial inner membrane protein involved in mitochondrial fusion. For studies of diseases such as optic atrophy, the mice described in this study would provide an excellent research tool.

### Study of Retinal Neuron Subtype Development

The expression of various combinations of transcription factors are required for specification of different populations of retinal neurons. Transcription factors required for generation of the various basic types of retinal neurons, such as rods or amacrine cells, have been identified; however, it is largely unknown how subtypes of retinal neurons are specified. The availability of transgenic labels that are expressed in specific subtypes of retinal neurons will simplify identification of such factors by allowing the isolation of specific subtypes of retinal neurons. Purified cell populations can also be used for gene expression profiling to obtain a better molecular definition of the differentiation state of the cells, to provide additional markers for studying the cells, and to better understand the molecular code of surface proteins that confer cell identity. Furthermore, being able to visualize specific cell types in vivo is a great boon to studies of retinal patterning and mosaic formation.

### Study of Clonal Proliferation and Lateral Migration

X-inactivation in the optic cup occurs by approximately embryonic day 9.5 (E9.5), during which retinal progenitor cells have not yet begun to differentiate into retinal neurons [13,14]. Production of retinal neurons occurs in a columnar clonal fashion, with a given population of retinal precursor cells giving rise to all populations of retinal neurons in a horizontal column [11,15]. Because the retina forms in a columnar fashion during development, with limited lateral migration, patches of YFP-positive and YFP-negative retina are observed resulting from either neural progenitors in which the X-Chromosome carrying the Mito-Z transgene is expressed or inactivated, respectively, making this line a useful marker of clonal proliferation (Figure 3B). The clonal columns generated by hemizygous female Mito-Z mice are not as obvious as those generated by other transgenic lines because YFP is not widely expressed in bipolar cells and photoreceptors, which do not migrate across the horizontal plane of the retina, while the amacrine and ganglion cells that YFP is expressed in do, making clonal borders less sharp [16]. The X-Chromosome location of the Mito-Z transgene will add another useful reagent, that can be used by itself or in combination with other X-Chromosome reporters such as a β-Gal reporter, for example by using both reporters in tandem to compare horizontal migration into and out of differentially labeled clonal columns [17]. The X-Chromosome insertion site of the transgene also simplifies maintaining Mito-Z mice, as male carries crossed to wild type females will sire only transgenic female and wild type male offspring.

In this report we characterize the retinal expression pattern of three transgenic mouse lines. These lines will be useful for a number of various studies, including analysis of mitochondrial trafficking, and will complement a similar line of mice that express of mitochondrial localized CFP in different populations of retinal neurons [18]. An increasing number of transgenic mouse lines offers the ability to label more retinal cell types, permitting an increasing amount of experimental flexibility and control [3,7,9,19]. The mice described here add value by labeling new subsets and by labeling mitochondria allowing new fields of study.

## Materials and methods

### Animal care and handling

Mice were used in accordance with protocols approved by the Animal Care and Use Committee at The Jackson Laboratory. The Jackson Laboratory is accredited by AAALAC. Animals were housed in PIV caging and given food and water ad libitum, and maintained on a 14 hour:10 hour light:dark cycle.

### Mouse strains

The following mouse strains were used in this study: Mito-R: Tg(Eno2-YFP/Cox8a)RRwb/J [8], Mito-Y: Tg(Eno2-YFP/Cox8a)YRwb/J (Jax stock number 007857) [8], Mito-Z: Tg(Eno2-YFP/Cox8a)/ZRwb (Jax stock number (007860) [8].

### Tissue Preparation and staining

Mice were transcardially perfused with PBS followed by perfusion with 4% paraformaldahyde buffered with PBS. Eyes were then fixed for an additional one hour in 4% paraformaldahyde buffered with PBS. Eyes were enucleated and sunk in 30% sucrose overnight and then frozen in cyropreservation media. Sections of retina were cut at 8 μm thickness with a cyrostat. Sections were rinsed in PBS and mounted with antifade or stained with antibodies.

Immunofluorescence was performed by blocking sections in PBS with 3% normal horse serum and 0.1% triton x-100, and then incubating sections overnight at 4°C with primary antibodies diluted in blocking buffer. Sections were then washed three times for ten minutes in PBS and incubated with secondary antibodies diluted in blocking buffer. Sections were washed three times for ten minutes in PBS, with DAPI included in the last wash, and mounted with antifade media. The following commercially available antibodies were used: mouse anti-nonphosphorylated neurofilament (1:200; Covance Research Products), rabbit anti-tyrosine hydroxylase (1:500 Invitrogen), goat anti-ChAT (1:500, Chemicon) and goat anti-BRN3b (1:200; Santa Cruz Biotechnology).

Whole retinas were prepared by fixing as described above. Retinas were then blocked in PBS, 3% normal horse serum and 0.4% triton X-100. Retinas were incubated in primary antibodies diluted in the blocking solution for four days at 4°C and then washed overnight in blocking buffer. Retinas were incubated overnight with secondary antibodies diluted in blocking buffer and washed in blocking buffer for an additional day and then mounted with antifade media. All images were collected on a Leica SP5 confocal microscope.

## Competing interests

The authors declare that they have no competing interests.

## Authors' contributions

PGF: Microscopy and writing of manuscript. RWB: Design of Mito-Y transgenic mice and editing of manuscript. All authors have read and approved the final manuscript.
